# Platelet-Derived Microparticles Bearing PF4 and Anti-GAGS Immunoglobulins in Patients with Sepsis

**DOI:** 10.3390/diagnostics10090627

**Published:** 2020-08-24

**Authors:** Maria Teresa Sartori, Chiara Zurlo, Maria Bon, Antonella Bertomoro, Raffaele Bendo, Irene Bertozzi, Claudia Maria Radu, Elena Campello, Paolo Simioni, Fabrizio Fabris

**Affiliations:** Department of Medicine DIMED, Padova University Medical School, 35100 Padova, Italy; mtsart@unipd.it (M.T.S.); chiara.zurlo.1@gmail.com (C.Z.); maria.bon@aulss2.veneto.it (M.B.); antonella.bertomoro@aopd.veneto.it (A.B.); raffaele.bendo@unipd.it (R.B.); irene.bertozzi@unipd.it (I.B.); claudiamaria.radu@unipd.it (C.M.R.); elena.campello@unipd.it (E.C.); paolo.simioni@unipd.it (P.S.)

**Keywords:** platelet, sepsis, platelet microparticles, anti-heparin/PF4 antibody

## Abstract

PF4 is a megakaryocyte-derived cationic chemokine that plays a part in innate immunity through its activity on the macrophages. In bacterial sepsis, PF4 binds to glycosaminoglycans (GAGs) on the surface of aerobic bacteria, giving rise to an antigenic complex that induces the early formation of anti-PF4 IgG-IgA-IgM. This triggers the immune response in patients receiving heparin therapy who develop heparin-induced thrombocytopenia (HIT). These antibodies have also been identified in patients with chronic Gram-negative infections. Given the complexity of this innate immune response network, our study on 45 patients with sepsis focused on the immune response mediated by platelet PF4. We analyzed the role of IgG-IgA-IgM against PF4-GAGs, and the presence of specific PF4-bearing platelet microparticles (PMPs). Anti-GAGs/PF4 IgG-IgA-IgM levels were significantly higher in septic patients than in control groups (healthy controls or acute patients without sepsis, *p* < 0.001). PF4-bearing PMP levels were only significantly higher in septic patients (*p* < 0.001). The occurrence of IgG-IgA-IgM against PF4-GAGs and PF4+ PMPs correlated with an improvement in patients’ sepsis. In conclusion, we demonstrated that, in the course of bacterial sepsis, platelet activation leads to the formation of specific PF4-bearing PMPs. These specific microparticles bind to polyanionic sequences on the surface of aerobic bacteria, giving rise to an antigenic complex that induces the early formation of IgG-IgA-IgM against PF4-GAGs as an innate immune response to infection.

## 1. Introduction

Platelets play a key role in immunity through receptor interaction with bacteria and other cells involved in innate immunity, releasing chemokines, inflammation mediators, antimicrobial proteins, and microparticles (MPs) [1]. MPs are small (0.1–1.0 μm) membrane vesicles constitutively released from the surface of cells (erythrocytes, white blood cells, platelets, and endothelial cells) after activation and apoptosis [2]. One important property of platelet-derived microparticles (PMPs) is signal transfer, as seen in sepsis and heparin-induced thrombocytopenia (HIT) [2,3]. PF4 is a platelet-specific heparin-binding protein that is abundant in platelet alpha granules from which it is secreted following platelet stimulation [4]. In bacterial sepsis, PF4 binds to glycosaminoglycans (GAGs) on the surface of aerobic bacteria, forming an antigenic complex that may induce early generation of anti-GAGs/PF4 IgA-IgG-IgM [5,6] as a defense mechanism, prompting phagocytosis and contributing to innate immunity [7] and autoimmunity [8]. Similarly to patients with HIT, these antibodies have also been found in patients with chronic Gram-negative infections [9,10]. The immune complexes resulting from the interaction between the Fc gamma RIIa region of platelets and the reticuloendothelial system activate and sequester platelets thus releasing PF4+PMPs via a specific modality, possibly mediated by the bacterial lipopolysaccharide.

Considering the complexity of the innate immune response network, we chose to focus on the immune response mediated by PF4 in a model of sepsis in adults, analyzing the role of anti-GAGs/PF4 IgA-IgG-IgM and the formation of PMPs.

In particular, we hypothesized the following: (a) in the course of bacterial sepsis, PF4 binds to polyanionic sequences on the surface of aerobic bacteria, forming an antigenic complex that induces early formation of IgG-IgA-IgM directed against PF4-GAGs complexes; and (b) the binding of IgA-IgG-IgM to PF4-GAGs activates platelets, prompting the generation of PF4+PMPs expressing PF4 on their surface with a specific modality.

## 2. Materials and Methods

### 2.1. Cases: Patients with Medical Sepsis

The study was conducted in compliance with the Declaration of Helsinki and all patients gave written consent before enrollment. The Ethics Committee for Clinical Trials of the Province of Padua (3419/AO/15) approved the study on 23 April 2015. We initially enrolled 45 patients (M/F: 1/1) consecutively admitted to our clinical ward at Padova University Hospital. Eleven patients were excluded from the study for lymphoproliferative disease (n.3); transfer to an intensive care unit (n.6); or death (n.2). Patients were classified as having sepsis or severe sepsis according to the Society of Critical Care Medicine (SCCM)/ European Society of Intensive Care Medicine (ESCIM) criteria [11].

The National Early Warning Score (NEWS) [12] and the Sequential Organ Failure Assessment (SOFA) [13] scores were used as stability indices for the evolution of sepsis. Patients with sepsis who had a Padua Prediction Score ≥ 4 [14] received thromboprophylaxis with low-molecular-weight heparin (LMWH).

### 2.2. Controls

After obtaining the informed consent, we enrolled 93 controls aged > 18 years, who were divided into two groups: (a) 65 healthy volunteers not previously exposed to heparin and (b) 28 patients hospitalized in our medical ward for acute condition without sepsis and treated for at least 5 days with prophylactic LMWH. Exclusion criteria for cases and controls were as follows: age < 18; failure to obtain informed consent; and presence of any lymphoproliferative disorders, HIV+, HCV+ or HP+, thrombocytopenia (platelets < 150 × 10^9^/L), immunosuppressive therapy, or history of severe infections in the previous 3 months.

### 2.3. Blood Sampling and Assays

Venous blood sampling was conducted with a 21G needle on admission (T0) for patients and controls, then in patients with sepsis 7 ± 2 days after admission (T1). At T0, before initiating any antibiotic therapy, samples were taken for microbiological cultures. Other analyses performed by standard methods included: white blood cell count (WBC), C reactive protein (CRP), procalcitonin, lactic acid, prothrombin time (PT), activated partial thromboplastin time (aPTT), fibrinogen, D-dimer, and antithrombin III (ATIII).

Anti-GAGs/PF4 IgG and IgA-IgG-IgM antibodies: the ELISA (enzyme-linked immunosorbent assay) methods from Immucor GTI Diagnostics, Inc. (Waukesha, WI, USA) were used to measure antibody levels, as described previously [15,16]. The antigen immobilized was PF4-CXCL4 complexed to polyvinyl sulfonate (PVS). The test results were expressed as absolute optical density (OD).

Circulating MPs were assayed by flow cytometry (Cytoflex, Beckman Coulter, Pasadena, CA, USA), as described previously [3,17]. Total MPs were identified by their dimensional features using beads of specific diameters with conformational characteristics resembling those of MPs (Megamix SSC-fluorescent beads [0.16, 0.24, 0.5, 0.9 and 3 μm]), and labeling them with monoclonal anti-annexin V conjugated with Fluorescein isothiocyanate (FITC- Bender Med Systems GmbH, Vienna, Austria).

Platelet derived microparticles (PMPs) were identified by co-labeling the anti-annexin V antibody with specific monoclonal antibodies to surface platelet antigen (anti-CD61 conjugated with PC5 (Beckman Coulter, Miami, USA), anti-PF4-CXCL4 conjugated with PE (R&D Systems, Inc., Minneapolis, USA) (PF4+ PMPs) and anti-CD62P conjugated with PC5 (Beckman Coulter, Miami, USA).

### 2.4. Statistical Analysis

Nominal variables were reported as number and frequency (%). Numerical variables were summarized by mean (standard deviation) or median (1st–3th quartiles). The X2 test was used to compare categorical variables. Comparison between continuous variables was performed by either Student’s *T*-Test or the Mann–Whitney U test where indicated.

The analysis of correlation between numerical variables was performed according to Spearman’s rank nonparametric correlation test. A two-tailed *p* value of less than 0.05 was considered significant. All statistical analyses were performed using the SPSS software (Statsoft Inc., Tulsa, OK, USA) version 25.

## 3. Results

There were significant differences between cases and controls without sepsis as regards infection indices, PT, D-dimer and heparin therapy (Table 1). The infectious agent was identified in 70% of patients with sepsis: 67% were Gram-negative, and 33% were Gram-positive bacteria. Patients with sepsis were treated with monotherapy antibiotic in 44% of cases, and combination antibiotic therapy in 56%. In the former, penicillins (18%) were used most often, followed by carbapenems, glycopeptides and tigecycline (6% each). In the latter, the most frequent associations were penicillins + fluoroquinolones (21%) and penicillins + glycopeptides (11%)

As reported in Table 2, patients with sepsis showed a significant reduction in inflammatory indices at T1, whereas hemostatic values and platelet count did not change. From the clinical standpoint, we observed a statistically significant reduction in NEWS and SOFA scores at T1 vs. T0.

**Figure 1:**
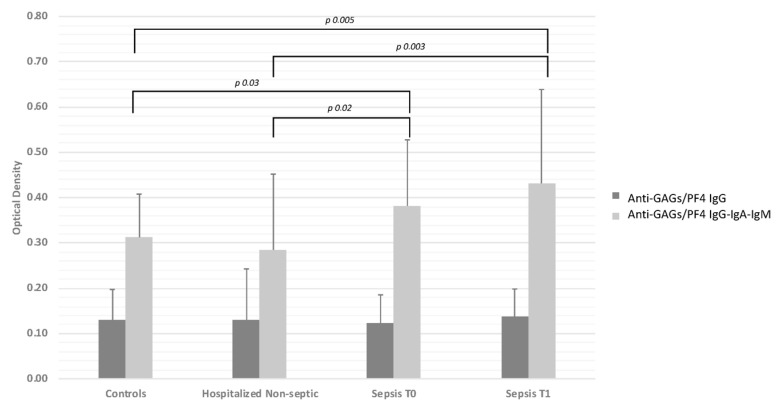
Titer of anti-glycosaminoglycans (GAGs)/PF4 IgG and anti-GAGs/PF4 IgG-IgA-IgM antibodies in healthy controls, patients with acute non-septic disease, and patients with sepsis, on admission (T0) and 7 ± 2 days afterwards (T1). Bars indicate mean ± standard deviation.

Median anti-GAGs/PF4 IgG levels rose from T0 to T1 in septic patients, though it was not statistically significant. Median OD values were similar in cases and both control groups. On the other hand, anti-GAGs/PF4 IgG-IgA-IgM levels were significantly higher in septic patients than in either control group (median OD values in patients with sepsis 0.35, 0.37 at T0 and T1, respectively vs. 0.30 in healthy subjects, and 0.22 in patients without sepsis) (Figure 1).

**Figure 2:**
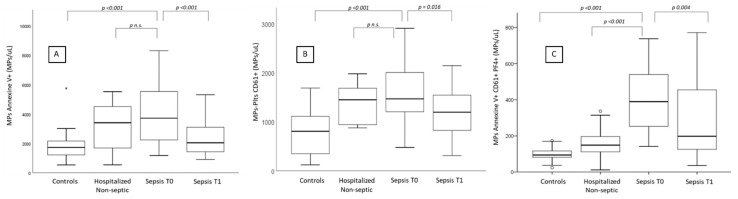
Numbers of microparticles (**A**: annexin V+), platelet-derived microparticles (**B**: annexin V+/CD61+) and platelet-derived microparticles bearing PF4 (**C**: annexin V+/CD61+/PF4+) in healthy controls, patients with acute disease without sepsis, and patients with sepsis, on admission (T0) and 7 ± 2 days afterwards (T1).

Compared with healthy subjects, the numbers of MPs bearing annexin V were significantly higher in both patients with sepsis—though significantly lower at T1 vs. T0 (*p* < 0.001)—and the control group of patients hospitalized for acute condition without sepsis (Table 3; Figure 2A). A similar downward trend from T0 to T1 was also observed as regards numbers of PMPs bearing CD61 in patients with sepsis (*p* < 0.001) (Table 4; Figure 2B).

Notably, PF4-bearing PMP numbers were markedly increased solely in septic patients with a significant difference with either control group. In the same patients with sepsis, CD62P-bearing PMP numbers were markedly increased and significantly correlated with PF4-bearing PMP (r = 0.564; *p* = 0.0014). Furthermore, PF4-bearing PMPs significantly decreased at T1 vs. T0 (*p* < 0.001) (Table 5; Figure 2C).

No significant correlations emerged between OD and MPs, or between OD and any of the clinical and biochemical parameters considered. Nor were there any correlations between OD and total MP or PF4+ PMP levels.

## 4. Discussion

Platelets play a key role not only in hemostasis, but also in innate immunity as pathogens may interact with platelets’ Toll-like receptors and adhesive glycoproteins, thus causing the release of platelet-specific chemokines such as PF4 [1]. The interaction between PF4 and bacteria also results in the production of specific natural antibodies that cross-react with heparin [5], and can sometimes cause HIT [8,9]. It has been suggested that, in presence of bacterial infections, PF4 binds to polyanionic sequences on the surface of aerobic bacteria to form an antigenic complex that induces an early formation of antibodies against PF4/GAGs [9].

To test this hypothesis, we prospectively studied the development of anti-PF4/GAGs antibodies in a cohort of 45 consecutive patients with sepsis and a control population (patients without sepsis and healthy volunteers). Patients with and without sepsis were older vs. healthy controls, though anti-PF4/GAGs antibodies are not age-related [5]. We enrolled young healthy subjects as a control cohort because they did not suffer from any disease and they not take drugs. Our cases presented mild sepsis, according to SOFA and NEWS scores, but inflammation indices improved significantly with antibiotic treatment during the study and platelet count remained normal.

Patients with sepsis were at high thrombotic risk because of a Padova Prediction Score (PPS) of 4 that frequently required thromboprophylaxis with subcutaneous LMWH [14]. As a control population, we subsequently enrolled healthy subjects and patients hospitalized for acute condition without sepsis who required thromboprophylaxis with LMWH, as LMWH therapy may influence the development of heparin-associated antibodies.

The pathogenic antibodies that cause HIT belong to the IgG class, whereas IgM appear to be linked to innate immunity [18].

Despite their exposure to heparin, none of our patients with sepsis or hospitalized controls had antibody titers against PF4/GAGs compatible with HIT. Mean OD values were below the clinical cut-off and comparable with those of the younger healthy controls, confirming that antibodies against PF4-GAGs are not age-related [5].

Sepsis did not appear to affect the titer of anti-PF4/GAGS IgG. Instead, we found high levels of anti-PF4/GAGS IgA-IgG-IgM antibodies in patients with sepsis, which increased after the onset of the infection. This behavior may be explained by an immune response mediated by early production of IgA or IgM antibodies, and no IgG. Alternatively, the production of IgG might have occurred later on, and therefore did not emerge during the short observation period of our study. Since primary immune response to heparin lacks IgM [19], the formation of anti-PF4/GAGS IgA-IgM may be linked to the interplay among bacteria, platelet PF4 and the innate immune system, as demonstrated by Maharaj [20]. This retrospective study, conducted with a tailored ELISA method, demonstrated the presence of IgA-IgG-IgM antibodies in a large cohort of patients with infections, and a low probability of HIT, regardless of any bacteremia and whether patients had Gram-negative or Gram-positive bacterial infections [19]. However, it bears noting that Pongas et al. reported a relatively low titer of anti-PF4/GAGS IgG in a cohort of patients with Gram-negative bacteremia, using an in-house ELISA [10].

The cell signals involved in intercellular communication are mediated at least in part by “extracellular vesicles”, such as exosomes, microparticles (MPs) and apoptotic bodies. Microparticles are vesicles with a diameter in the range of 0.1 to 1 µ, released from the membranes of various cell types [2]. Platelet-derived microparticles (PMPs) are the largest subgroup in human plasma, accounting for 70–90% of all circulating MPs [21]. They are formed mainly during platelet activation and express various platelet and plasma antigens on their surface. The level of circulating PMPs increases in many pathological conditions associated with inflammation, activation of the coagulation cascade, and sepsis [22,23]. In a recent study, we demonstrated for the first time the presence of PMPs expressing PF4 in patients with HIT derived from activated CD62P-positive PMPs and hypothesized that this mechanism was secondary to platelet activation [3].

The formation of MPs and PMPs during sepsis has been described previously and we were able to confirm that cellular and platelet activation in patients with sepsis leads to the formation of MPs and PMPs, which are probably linked to inflammation, rather than secondary to bacterial infection. In fact, numbers of MPs and PMPs were also higher in patients hospitalized with acute condition without sepsis [24].

PF4 is a strongly cationic chemokine packaged inside platelet alpha-granules, and released after platelet activation [25,26] so it is hardly surprising that it should be found on the surface of PMPs as well as other platelet antigens. However, the presence of PF4-bearing PMPs appears to correlate specifically with platelet activation secondary to bacterial infection, since we found markedly elevated numbers of PF4+ PMPs solely in patients with sepsis in whom CD62P+PMPs were also increased.

In conclusion, our findings suggest that platelet activation in the course of bacterial sepsis leads to the formation of specific PF4-bearing PMPs, which may bind to polyanionic sequences on the surface of aerobic bacteria, forming an antigenic complex that induces early formation of IgA-IgG-IgM against PF4 as part of the innate immune response.

## Figures and Tables

**Table 1:** Main features of patients with sepsis and control patients without sepsis.

	Patients with Sepsis	Control Patients without Sepsis	*p*
**Gender M/F n (%)**	17/17 (50%/50%)	10/18 (36%/64%)	ns
**Age in Years (M ± SD)**	74 ± 14.8	75 ± 12	ns
**LMWH IU/day (M ± SD)**	4000 ± 800	6769 ± 4563	<0.05
**WBC (×10^9^/L)**	11.46 ± 5.22	7.35 ± 2.19	<0.001
**N (×10^9^/L)**	9.09 ± 5.05	4.99 ± 2.13	<0.001
**Platelets (×10^9^/L)**	233 ± 113	223 ± 67	ns
**CRP (mg/L)**	175.4 ± 101	11.4 ± 10.1	<0.001
**Procalcitonin (ng/L)**	17.3 ± 37	0.57 ± 0.54	0.015
**PT (%)**	58.1 ± 23.7	85 ± 19.1	<0.001
**aPTT (s)**	30.4 ± 8.25	28.6 ± 4.23	ns
**D-dimer (ng/mL)**	873 ± 887.5	289.9 ± 201	0.046

LMWH: low molecular weight heparin; WBC: white blood cells; N: neutrophils; CRP: C-reactive protein; PT: prothrombin time; aPTT: partial thromboplastin time.

**Table 2:** Clinical findings over time in patients with sepsis.

	T0	T1	*p*
**WBC (×10^9^/L)**	11.46 ± 5.22	7.81 ± 3.95	<0.05
**N (×10^9^/L)**	9.09 ± 5.05	5.46 ± 3.73	<0.05
**Platelets (×10^9^/L)**	233 ± 113	266 ± 120	0.3
**CRP (mg/L)**	175.4 ± 101	68.4 ± 63	<0.05
**Lactic Acid (mmol/L)**	3.3 ± 0.8	1.1 ± 0.4	<0.05
**Procalcitonin (ng/L)**	17.3 ± 37	1.7 ± 2.2	<0.05
**PT (%)**	58.1 ± 23.7	58.8 ± 18.2	0.9
**aPTT (s)**	30.4 ± 8.25	28.18 ± 4.5	0.3
**Fibrinogen (mg/dL)**	5.8 ± 1.9	5.1 ± 0.7	0.3
**AT III**	68.2 ± 15.2	77.1 ± 16.2	0.2
**D-dimer (ng/mL)**	943.4 ± 89.5	907.4 ± 55.9	0.9
**NEWS score**	5.09 ± 1.17	1.1 ± 1.63	<0.05
**SOFA score**	9.47 ± 2.09	0.73 ± 0.66	<0.05

WBC: white blood cells; N: neutrophils; CRP: C-reactive protein; PT: prothrombin time; aPTT: partial thromboplastin time; AT: antithrombin; NEWS: National Early Warning Score; SOFA: Sequential Organ Failure Assessment.

**Table 3:** Numbers of annexin V+ microparticles in patients with sepsis, on admission (T0) and at 7 ± 2 days afterwards (T1), and in control patients with acute disease without sepsis, and healthy controls.

	Mean ± SD	Median	1st Quartile	3rd Quartile	*p*
**Sepsis T0**	3935 ± 1937	3251	2258	5516	
**Sepsis T1**	2314 ± 1119	2044	1449	3024	<0.001 (vs. T0)
**Non-Septic Control Patients**	3071 ± 1828	2388	1319	4832	NS
**Healthy Controls**	1797 ± 904	1728	1226	2147	<0.001 (vs. T0)

**Table 4:** Levels of platelet-derived microparticles (annexin+/CD61+) in patients with sepsis, on admission (T0) and at 7 ± 2 days afterwards (T1), and in control patients with acute non-septic disease, and healthy controls.

	Mean ± SD	Median	1st Quartile	3rd Quartile	*p*
**Sepsis T0**	1566 ± 534	1468	1205	2005	-
**Sepsis T1**	1163 ± 188	1197	825	1544	<0.016 (vs. T0)
**Non-Septic Control Patients**	1375 ± 402	1448	955	1595	NS
**Healthy Controls**	759 ± 437	807	355	1110	<0.001 (vs. T0)

**Table 5:** Levels of platelet-derived microparticles (annexin+/CD61+/PF4+) in patients with sepsis, on admission (T0) and at 7 ± 2 days afterwards (T1), and in control patients with acute non-septic disease, and healthy controls.

	Mean ± SD	Median	1st Quartile	3rd Quartile	*p*
**Sepsis T0**	1471 ± 414	398	279	562	-
**Sepsis T1**	282 ± 195	198	127	444	<0.004 (vs. T0)
**Non-Septic Control Patients**	86 ± 31	93	63	97	<0.001 (vs. T0)
**Healthy Controls**	98 ± 34	94	81	116	<0.001 (vs. T0)
